# Genomic reconstruction of transcriptional regulatory networks in lactic acid bacteria

**DOI:** 10.1186/1471-2164-14-94

**Published:** 2013-02-12

**Authors:** Dmitry A Ravcheev, Aaron A Best, Natalia V Sernova, Marat D Kazanov, Pavel S Novichkov, Dmitry A Rodionov

**Affiliations:** 1 Sanford-Burnham Medical Research Institute, La Jolla, CA 92037, USA; 2 A.A. Kharkevich Institute for Information Transmission Problems, Russian Academy of Sciences, Moscow 127994, Russia; 3 Department of Biology, Hope College, Holland, MI, 49423, USA; 4 Lawrence Berkeley National Laboratory, Berkeley, CA, 94710, USA

**Keywords:** Transcriptional regulatory network, Comparative genomics, Carbohydrate metabolism, Lactobacillaceae, Streptococcaceae, Lactic acid bacteria, Regulon, Transcription factor

## Abstract

**Background:**

Genome scale annotation of regulatory interactions and reconstruction of regulatory networks are the crucial problems in bacterial genomics. The *Lactobacillales* order of bacteria collates various microorganisms having a large economic impact, including both human and animal pathogens and strains used in the food industry. Nonetheless, no systematic genome-wide analysis of transcriptional regulation has been previously made for this taxonomic group.

**Results:**

A comparative genomics approach was used for reconstruction of transcriptional regulatory networks in 30 selected genomes of lactic acid bacteria. The inferred networks comprise regulons for 102 orthologous transcription factors (TFs), including 47 novel regulons for previously uncharacterized TFs. Numerous differences between regulatory networks of the *Streptococcaceae* and *Lactobacillaceae* groups were described on several levels. The two groups are characterized by substantially different sets of TFs encoded in their genomes. Content of the inferred regulons and structure of their cognate TF binding motifs differ for many orthologous TFs between the two groups. Multiple cases of non-orthologous displacements of TFs that control specific metabolic pathways were reported.

**Conclusions:**

The reconstructed regulatory networks substantially expand the existing knowledge of transcriptional regulation in lactic acid bacteria. In each of 30 studied genomes the obtained regulatory network contains on average 36 TFs and 250 target genes that are mostly involved in carbohydrate metabolism, stress response, metal homeostasis and amino acids biosynthesis. The inferred networks can be used for genetic experiments, functional annotations of genes, metabolic reconstruction and evolutionary analysis. All reconstructed regulons are captured within the *Streptococcaceae* and *Lactobacillaceae* collections in the RegPrecise database (http://regprecise.lbl.gov).

## Background

Regulation of gene expression in response to external and internal stimuli is a crucial mechanism for adaptation of microorganisms to changes of environmental conditions and intracellular states. In Bacteria, regulation of gene expression at the transcriptional level is usually mediated by transcription factors (TFs) that recognize their cognate TF-binding sites (TFBSs) in the promoter regions of regulated genes. A set of target genes under direct control of a certain TF is defined as a regulon. All regulons in a single organism establish the transcriptional regulatory network (TRN), a fine-tuned system for complex regulation of gene expression in response to environmental changes and physiological needs of the cell.

Reconstruction of TRNs in bacterial genomes involves identification of regulatory interactions between target operons and TFs that requires genome-wide definition of all respective TFBSs. Various approaches for TRN reconstruction have been developed including traditional bottom-up genetic methods [1-3] and new top-down techniques based on the large-scale expression data [4] and/or automated inference of TFBS motifs [5-8]. On the other hand, the growing number of available complete genomic sequences opens opportunities for comparative genomic analysis of transcriptional regulation and subsequent TRN inference (reviewed in [9,10]). This analysis can be efficiently used for both propagation of known regulons to previously uncharacterized organisms and for *ab initio* discovery of novel regulons. Combination of the comparative genomic-based regulon reconstruction with other genome context analysis techniques and multiple available experimental datasets define a knowledge-driven approach for reconstruction of TRNs in a set of related bacterial genomes. This approach has been successfully used for TRN inference in two groups of related genomes including 16 *Shewanella* species [11] and six species from the *Staphylococcaceae* family [12]. The comparative genomics approach not only allowed us to reconstruct TRNs in multiple genomes but also resulted in prediction of functions for previously uncharacterized genes.

In this study, we applied the knowledge-driven comparative genomic approach for reconstruction of TRNs in lactic acid bacteria belonging to the *Lactobacillales* order of the Firmicutes phylum. In spite of the large number of complete genomes and huge economic impact of this group (reviewed in [13-15]), most studies of gene regulation in these bacteria are limited to individual regulons in some model species; large-scale TRN reconstructions were attempted for *Lactobacillus plantarum*[16] and *Lactococcus lactis*[17]. On the other hand, availability of complete genomic sequences and multiple experimental data on gene regulation provide an opportunity for application of comparative genomic-based techniques for reconstruction of TRNs in lactic acid bacteria. To reconstruct genome-wide TRNs in the set of 30 *Lactobacillales* genomes we used a modified approach with three innovations. First, we subdivided the analyzed group of genomes into two phylogenetically distinct groups: the *Streptococcaceae* group including species from the *Streptococcus* and *Lactococcus* genera, and the *Lactobacillaceae* group that also includes two closely-related genomes from the *Leuconostocaceae* family. Second, to compose the initial training sets of TF-regulated genes we used the available experimental data on transcriptional regulation from 14 model species. Third, the coordinated reconstruction of a large number of TF regulons was carried out by a community of annotators using the RegPredict platform [18] with subsequent curation and quality control. Using this combined approach we reconstructed regulons for 102 orthologous TFs, including 47 novel regulons predicted for the first time in this study and awaiting further experimental validation.

## Results and discussion

We selected a set of 30 complete genomes in the *Lactobacillales* order for TRN reconstruction. Based on the phylogenetic species tree (Additional file 1) all studied genomes were divided into two groups called the *Streptococcaceae* and *Lactobacillaceae*. The *Streptococcaceae* family includes 13 *Streptococcus* spp. and 2 strains of *Lactococcus lactis*. The second group includes 13 genomes from the *Lactobacillaceae* family and 2 genomes form the *Leuconostocaceae* family that are phylogenetically close to each other [19].

### Repertoire of TFs in *Lactobacillales* genomes

To estimate the scale and diversity of the TF mediated regulatory networks in the *Lactobacillales* genomes, we performed a genetic census of their putative DNA-binding TFs using similarity search and the existing prokaryotic TF compilations (Additional files 2, 3). The total number of putative TFs varies broadly within the *Lactobacillales* genomes, from ~60 in *S. thermophilus* and *L. helveticus* to ~240 in *L. plantarum* and ~150 in *S. gallolyticus* (Additional file 4).

The putative TFs identified in the *Lactobacillales* are distributed between 49 protein families and about 90% of these TFs belong to 24 major families with at least two representatives per genome. The largest number of TF representatives was observed for the Xre family (298 TFs total, ~19 TFs per genome). Among other large families with more than 4 TFs per genome are the TetR, GntR, MarR, OmpR, LacI, LysR, MerR and AraC families. Comparison of TF repertoires between the two *Lactobacillales* groups reveals 42 TF families that have representatives in both groups. Among the lineage-specific TF families, three families (CodY, PF04394, YobV) are present only in the *Streptococcaceae*, whereas four families (LexA, SdaR, SfsA, ComK) are unique for the *Lactobacillaceae*. Interestingly, the LexA and CodY regulators are both present in the *Staphylococcaceae, Bacillaceae,* and *Enterococcaceae* families of Firmicutes, suggesting the family-specific loss of these TFs and their regulons in the *Streptococcaceae* and *Lactobacillaceae* families, respectively.

**Figure 1:**
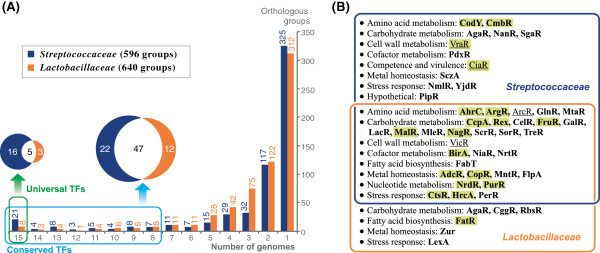
**Distribution of TF orthologous groups in studied genomes.** (**A**) Distribution of TF orthologous groups in genomes. Conserved TFs are present in more than half analyzed genomes within the lineage. Universal TFs are present in all genomes of the lineage. (**B**) Examples of functional annotations for conserved TFs. TFs for which regulons were reconstructed are shown in bold, other TFs are underlined. TFs universally conserved in at least one lineage of the *Lactobacillales* are highlighted in green.

The entire set of 3445 TFs identified in 30 studied genomes was broken into 596 orthologous groups in the *Streptococcaceae* and 640 orthologous groups in the *Lactobacillaceae* (Additional files 2, 3). The numbers of universal TFs present in all analyzed *Streptococcaceae* and *Lactobacillaceae* genomes are 21 and 8, respectively (Figure 1). At that, only 5 regulators are shared between these two groups of universal TFs including the global regulator for catabolite repression CcpA [20,21], the copper uptake regulator CopR [22], the purine biosynthesis regulator PurR [23], the redox control global regulator Rex [12], and the aminosugar utilization regulator NagR [24]. In each taxonomic group we defined all TFs that are present in more than half of the analyzed genomes as conserved TFs. Among the conserved TFs, 47 are shared by both studied lineages, whereas the *Streptococcaceae* and *Lactobacillaceae* groups have respectively 22 and 12 conserved TFs that are lineage-specific. The regulators of carbohydrate and amino acid metabolism form the most populated functional group of conserved TFs (Figure 1).

The studied two taxonomic groups of lactic acid bacteria demonstrate different distributions of orthologous TFs. The fractions of universal and conserved TFs are significantly higher in the *Streptococcaceae* than in the *Lactobacillaceae*. On the other hand, the *Lactobacillaceae* genomes are equipped by a confidently higher fraction of sporadically distributed TFs that are present in 2 to 6 genomes. Thus, the *Lactobacillaceae* are characterized by higher variability of TFs orthologous groups than the *Streptococcaceae* that is in correspondence with the larger phylogenetic distances between species in the *Lactobacillaceae* family (Additional file 1).

### Reconstruction of regulons in two lineages of Lactobacillales

**Figure 2:**
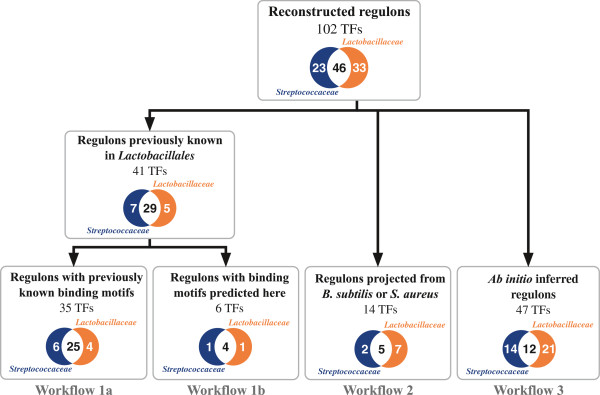
**Workflows used for regulon inference.** Venn diagrams show numbers of regulons reconstructed by every approach: white background, regulons shared by both lineages; blue background, *Streptococcaceae* specific regulons; red background, *Lactobacillaceae* specific regulons.

A comparative genomic approach implemented in the RegPredict Web server [18] was applied for regulon inference in the *Streptococcaceae* and *Lactobacillaceae* groups of genomes. Totally, 102 orthologous TF regulons were reconstructed in the studied genomes (Additional file 5). Initially we collected the published experimental data on transcriptional regulation in model *Lactobacillales* species (Additional file 5). Depending on the availability of experimental data, we applied three different workflows for regulon inference: (1) expansion and projection of TF regulons previously characterized in *Lactobacillales*, (2) the reconstruction of regulons for TFs that have orthologs previously characterized in *B. subtilis* or *S. aureus*, and (3) *ab initio* inference of regulons for previously uncharacterized TFs (Figure 2).

A significant number of the studied *Lactobacillales* genomes, including eight *Streptococcus*, two *Lactococcus* and four *Lactobacillus* species, were considered as model species with previously characterized TFs and regulons (Additional file 5). By using workflow 1, we propagated the previously established regulatory interactions for 41 TFs in the model *Lactobacillales* species, and predicted new regulon members by the comparative genomics approach. Using workflow 2, we inferred regulons for twelve TFs that have orthologs previously experimentally investigated in *B. subtilis* and two TFs that were previously studied in *S. aureus.* Finally, using workflow 3, we predicted and described 47 novel TF regulons (Figure 2). Thus, all TF regulons studied in this work are either entirely predicted by computational analysis regulons or partially predicted regulons with some regulatory interactions supported by experimental data in model species.

**Figure 3:**
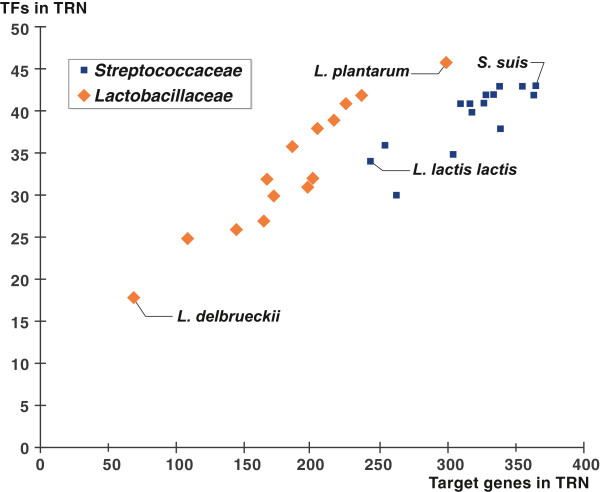
**Scatter plot for numbers of TFs and target genes in studied genomes.** Genomes with minimal and maximal numbers of target genes in each lineage are signed.

The resulting set of reconstructed regulons varies drastically between the individual genomes (Table 1). The average number of reconstructed TF regulons per genome is 35.8, whereas the minimal and maximal numbers are 18 and 46 that were inferred in *L. delbrueckii* and *L. plantarum*, respectively (Figure 3). An average regulatory network of the reconstructed TF regulons includes ~250 genes per genome. The minimal number of genes in a reconstructed TRN was observed in *L. delbrueckii* (69 genes), whereas the maximal number of regulated genes in a network was in *S. suis* (366 genes). In summary, the number of regulated genes in the reconstructed TF regulons is significantly higher in the *Streptococcaceae* genomes. However, *L. plantarum* from the *Lactobacillaceae* group has numbers of TFs and target genes in the reconstructed TRN comparable with *Streptococcaceae*. The observed variability in reconstructed TRNs of studied microorganisms can be explained by multiple factors including the diversity of their respective ecological niches and nutrient availability, and different metabolic capacities of individual species.

**Table 1:** Statistics for reconstructed regulons in studied genomes

**Genome**		**TFs**	**Target genes**	**Target operons**	**Regulatory interactions**
**Streptococcaceae**	*L. lactis cremoris* SK11	36	255	125	130
	*L. lactis lactis* Il1403	34	244	128	138
	*S. thermophilus* CNRZ1066	30	263	125	141
	*S. agalactiae* 2603 V/R	38	340	159	186
	*S. uberis* 0140 J	42	330	156	183
	*S. equi* MGCS10565	42	334	143	167
	*S. dysgalactiae* GGS_124	43	356	160	189
	*S. pyogenes* M1 GAS	40	319	150	180
	*S. gallolyticus* UCN34	41	328	167	199
	*S. mutans* UA159	41	317	147	173
	*S. suis* 05ZYH33	43	366	145	173
	*S. mitis* B6	35	305	148	174
	*S. pneumoniae* TIGR4	42	365	167	206
	*S. gordonii* CH1	41	312	167	194
	*S. sanguinis* SK36	43	339	163	191
	**Total**	**591**	**4773**	**2250**	**2624**
	**Non-overlapping**^**1**^	**69**	**779**	**397**	**470**
**Lactobacillaceae**	*L. sakei* 23 K	36	186	92	106
	*L. casei* ATCC 334	41	226	110	120
	*L. rhamnosus* GG	42	237	106	116
	*L. delbrueckii* ATCC BAA-365	18	69	36	37
	*L. acidophilus* NCFM	27	165	80	90
	*L. helveticus* DPC 4571	21	91	53	55
	*L. johnsonii* NCC 533	26	145	78	87
	*P. pentosaceus* ATCC 25745	38	205	97	111
	*L. brevis* ATCC 367	39	217	112	128
	*L. plantarum* WCFS1	46	299	147	170
	*L. fermentum* IFO 3956	30	172	85	101
	*L. reuteri* JCM 1112	32	167	83	96
	*O. oeni* PSU-1	25	109	59	70
	*L. mesenteroides* ATCC 8293	32	202	89	103
	*L. salivarius* UCC118	31	198	86	96
	**Total**	**484**	**2688**	**1313**	**1486**
	**Non-overlapping**^**1**^	**79**	**539**	**289**	**328**

^1^Numbers of orthologous groups of TFs, genes and operons in lineage.

### Classification of reconstructed regulons by taxonomic distribution

Distribution and properties of 102 TF regulons in 30 genomes of the *Lactobacillales* are summarized in Additional file 5. In each of the two taxonomic groups, the *Streptococcaceae* and *Lactobacillaceae*, we defined TF regulons that are universal (*i.e.* present in all 15 genomes) and the remaining regulons with a mosaic distribution in the analyzed genomes (Table 2). Only five regulons including the global regulons CcpA and Rex were found to be universally conserved in both lineages. Ten regulons that are universal in the *Streptococcaceae* have orthologs with mosaic distribution in the *Lactobacillaceae*. In contrast, no TF regulons appeared in the group of regulons that are universal in the *Lactobacillaceae* but mosaic in the *Streptococcaceae*. A large set of 31 TF regulons that are mosaic in both lineages contains regulons controlling different sugar utilization pathways.

**Table 2:** **Distribution of TFs with reconstructed regulons for orthologous TFs in *Streptococcaceae *and *Lactobacillaceae *genomes**

**TF type**^**1**^		**TF number**	**Examples**
**Streptococcaceae**	**Lactobacillaceae**		
Universal	Universal	5	CcpA, CopR, NagR, PurR, Rex
Universal	Mosaic	10	AdcR, ArgR, BirA, CtsR, FabT, FruR, GlnR, HrcA, MalR, NrdR
Mosaic	Universal	0	n/a
Mosaic	Mosaic	31	CcpB, CelR, FucR, GalR, GutR, LacR, MdxR, MleR, MntR, MtaR, MtlR, MurR, NiaR, NrtR, PadR, PerR, PflR, ScrR, TagR, TreR, UxuR
Universal	―	3	CmbR, CodY, PipR
Mosaic	―	20	AgaR, AlsR, CelQ, HomR, NanR, NmlR, PdxR, RegR, Rgg, RgrA, RliC, SczA, SgaR
―	Universal	1	LexA
―	Mosaic	32	AguR, AraR, CggR, DeoR, ExuR, FatR, HxlR, IolR, NihR, RbsR, RpiR, SdaR, XylR, Zur

Numbers of regulons for orthologous TFs in *Streptococcaceae* and *Lactobacillaceae* are shown. ^1^ ‘Universal’ regulons are present in all 15 studied genomes of the group; ‘Mosaic’ regulons are present in group but in less than 15 genomes; ‘―’ means the absence of regulons in this group.

The remaining TF regulons reconstructed in this work are present only in a single taxonomic group. Among 23 TF regulons present solely in the *Streptococcaceae*, the CmbR, CodY and PipR regulons are universal, whereas the others have a mosaic distribution in 15 analyzed genomes. The SOS response regulon LexA is only linage-specific regulon that was identified as universal in the *Lactobacillaceae*, whereas the remaining 20 lineage-specific TF regulons have a mosaic distribution in this lineage.

In conclusion, 23 TF regulons are specific for the *Streptococcaceae*, 33 regulons are present only in the *Lactobacillaceae* and 46 regulons have orthologs in both lineages. Thus, among the reconstructed regulons, the *Streptococcaceae* group has significantly larger number of universal TF regulons in comparison with the *Lactobacillaceae* group.

### Classification of reconstructed regulons by TF protein families

The reconstructed regulons are controlled by TFs from 31 protein families. The mostly represented TF family in the obtained set of regulons is the LacI family (26 regulons). As expected, all LacI family regulons reconstructed in this work controls different carbohydrate utilization pathways. Another broadly represented TF family is GntR family (13 regulons) that control carbon and amino acid metabolisms and resistance to toxic compounds. TFs from the TetR and MarR families (7 and 6 reconstructed regulons, respectively) regulate genes involved in environmental adaptation, multidrug and heavy metal resistance and fatty acids metabolism. The reconstructed regulons from the RpiR family control various carbohydrate utilization pathways (5 regulons), whereas the predicted AguR regulon controls the agmatine utilization pathway. The BglR and DeoR families of TFs include respectively 6 and 5 regulators that control carbohydrate catabolism. Three regulons operated by TFs from the LysR family regulate the cysteine/methionine metabolism, whereas one regulon (MleR) controls the malonate utilization pathway.

### Classification of reconstructed regulons by function

The functional gene content was assessed for each reconstructed TF regulon to tentatively predict its possible biological function and molecular effector. Metabolic reconstruction of the respective biochemical pathways and prediction of functions of co-regulated genes were performed using the subsystem-based approach implemented in the SEED genomic platform [25,26]. The inferred functional annotations of genes constituting regulons were captured in the RegPrecise database [27] within the taxonomic collections of regulons for the *Streptococcaceae* and *Lactobacillaceae* groups (http://regprecise.lbl.gov/RegPrecise/collections_tax.jsp).

Overall, the reconstructed regulons were classified into 8 functional groups (Additional file 5). The largest group counts 56 regulons for carbohydrate and central carbon metabolism. Three other functional groups of regulons are involved of stress response (11 regulons), metal homeostasis (9 regulons) and amino acid metabolism (8 regulons). Small numbers of regulons were reconstructed for cofactor metabolism (4 regulons), nucleotide metabolism (3 regulons) and fatty acid metabolism (2 regulons). Additionally, 9 reconstructed TF regulons contain genes with unknown or hypothetical functions, and thus their specific functional roles and effectors remain unknown. Functional content for the selected subset of reconstructed regulons is briefly described below.

#### Carbohydrate metabolism

Carbohydrates comprise a key source of carbon and energy for a variety of microorganisms. Genomes from the *Lactobacillales* order encode a large number of sugar catabolic pathways and most of these pathways have a mosaic distribution in individual species [28]. This diversity of sugar catabolic pathways is matched by a large number of regulatory systems that allow sugar-specific induction of expression of these pathways.

Here we reconstructed 56 TF regulons that control the sugar and central carbon metabolism in the *Lactobacillales*. In addition to 19 sugar metabolism regulons that were previously characterized experimentally in model *Lactobacillales* species, 6 regulons were reconstructed by projection from *B. subtilis* or *S. aureus* and 31 novel regulons were predicted for the first time in this work (Additional file 5). Analysis of available experimental data revealed 114 previously known regulatory interactions involving the sugar metabolism regulators in model *Lactobacillales* organisms. Using comparative genomics, we were able to significantly expand the sugar metabolism regulatory subnetwork. As an example, the previously known regulator of hyaluronidase RegR in *S. pneumoniae*[29] was predicted to have an expanded regulon of 12 additional genes involved in the hyaluronate utilization that is conserved in six *Streptococcaceae* genomes (Additional file 6).

About 10 new regulatory interactions per genome were predicted for carbohydrate metabolism genes using the projection of known regulons from *B. subtilis* and *S. aureus*. For example, CcpN in *B. subtilis* was previously described as a regulator of the gluconeogenesis genes *gapB* and *pckA*[30]. The comparative genomics reconstruction of the CcpN regulon revealed the pyruvate phosphate dikinase *ppdK*, the *ccpN* gene and the fructose biphosphatase *fbp* as novel members of the CcpN regulon in the *Lactobacillales* (Additional files 6, 7). Thus, the novel CcpN regulon in the *Lactobacillales* have a set of target genes that is completely different from the known CcpN regulon in *B. subtilis*, although in both lineages it controls the gluconeogenesis pathway.

The reconstructed regulatory network includes 52 TFs that control 38 peripheral sugar utilization pathways, and 10 of these pathways are controlled by more than one TF. For instance, we reconstructed 5 different TF regulons for maltose and maltodextrin utilization pathway and 3 TF regulons for the sucrose catabolism. Two different TF regulons per one sugar metabolic pathway were described for pathways involved in utilization of ascorbate, cellobiose, gluconate, lactose, N-acetylgalactosamine, ribose and trehalose. The observed redundancy in sugar-specific TFs is explained by (i) non-orthologous replacements of TFs for the same pathway in different genomes, and (ii) existence of alternative pathway variants and multiple paralogs regulated by different TFs in the same genome. For example, two maltose/maltodextrin ABC transporters are controlled by two non-orthologous TFs from the LacI family in the *Streptococcaceae* (Additional file 8). The *malEFG* operon is always regulated by MalR protein. The *malXCDA* operon is regulated by MalR in four *Streptococcus* spp. and under control of MalR2 in three other *Streptococcus* spp.

Three other sugar utilization pathways in the *Streptococcus* spp. are equipped by a redundant set of catabolic genes controlled by multiple non-orthologous regulators. SgaR and SgaR2 from the BglG family control the ascorbate utilization. The GntR family regulators AgaR and AgaR2 were predicted to control the N-acetylgalactosamine utilization gene loci. The cellobilose utilization genes are regulated by CelR from the BglG family [31] and CelQ from the ROK family [32]. These functionally redundant sets of TF regulons indicate a complex evolutionary history of the sugar utilization subsystems in Firmicutes.

#### Stress response

We reconstructed totally 11 TF regulons involved in various stress responses and drug resistance. These include the CtsR, HrcA, NmlR, PadR and PerR regulons that were previously experimentally described in at least one model *Lactobacillales* genome. The SOS response regulon LexA and multidrug resistance regulon YtrA were projected using the previous knowledge of orthologous regulons in *B. subtilis*[33,34]. Overall, we predicted 13 target operons in the *Lactobacillaceae* LexA regulon including some novel functions (e.g. *parEC*, *addBA*, *nrnA*).

The Fur family regulator PerR was previously studied in *S. pyogenes* and *S. suis*, where it co-regulates genes involved in peroxide stress response and manganese transport [35,36]. Here we predicted some new members of the PerR regulon involved in iron transport (*fhuADBG*, *feoAB*, *fatDCAB*) and iron-sulfur cluster biosynthesis (*sufCDSEB*). The latter iron-sulfur cluster biosynthesis operon was previously identified by us as a novel member of the PerR regulon in the *Staphylococcaceae* family [12]. Interestingly, we also predicted that in some *Streptococcus* spp., PerR controls expression of another TF for manganese homeostasis, MntR, and these two regulators form a potential cascade.

#### Metal homeostasis

The group of reconstructed regulons for metal homeostasis includes 9 TF regulons. Starting from 24 regulatory interactions previously described in 5 known regulons for model *Lactobacillales* organisms, we expanded and projected the AdcR, CopR, FlpA, MntR and SczA regulons resulting in prediction of several new regulatory interactions in the genomes. For instance, we predicted regulation of genes encoding manganese (*mntH*) and nickel (*nikABCDE*) transporters, heavy metal-transporting ATPase (*pmtA*), and *mntR* gene by the manganese homeostasis regulon MntR in the *Streptococcaceae*.

In contrast to the AdcR regulon for control of zinc homeostasis in the *Streptococcaceae*, the *Lactobacillaceae* species utilize the typical zinc-responsive regulator Zur from the Fur family. The Zur regulon in the *Lactobacillaceae*, which was reconstructed by projection from the *B. subtilis* Zur regulon, includes the zinc transporter *znuABC*, and the *zur* and *rpsN2* genes, the latter encoding a paralog of the ribosomal protein S14.

Two novel TFs from the ArsR family analyzed in the *Lactobacillaceae* are distantly related to the CzcR regulator of cobalt-zinc-cadmium resistance from *Pseudomonas aeruginosa*[37]. The reconstructed CzcR1 and CzcR2 regulons were predicted to control different sets of genes involved in heavy metal resistance. Another novel regulon, termed NihR (nickel homeostasis regulator), was predicted by its conserved co-localization with the *nikMQO* genes encoding a nickel transport system. The predicted NihR regulon also includes the *nihR* and *nixA* genes, the latter encoding a high-affinity nickel permease (Additional file 7).

#### Amino acids metabolism

Among 8 reconstructed TF regulons for control of amino acid metabolism, seven regulons have been previously described in at least one model *Lactobacillales* species. For these TF regulons, we projected 56 regulatory interactions to additional genomes and predicted ~25 novel regulatory interactions per genome. First, we predicted the MtaR- and CmbR-dependent regulation of the *mmuMP* operon involved in S-methylmethionine utilization in the *Streptococcaceae*. Second, we identified multiple new members of the nitrogen metabolism regulon GlnR in both lineages including the glutamine transporter *gluQHMP*, the arginine catabolism genes *arcABC* and the aspartate-ammonia ligase *asnA*. A novel amino acid metabolism regulon that was inferred for the first time in this work is operated by a RpiR-like regulator, termed AguR (agmatine utilization regulator), which was found to be co-localized with agmatine utilization genes in the *Lactobacillaceae* genomes.

#### CRISPR-Cas genes under the control of global regulators

CRISPR-Cas is a recently discovered prokaryotic RNA-based system for adaptive immunity for defense against phages, plasmids and other mobile genetic elements [38,39]. Previously, the expression of CRISPR-Cas genes has been shown to be regulated by different global TFs, such as H-NS, Lrp, LeuO [40,41] and Crp [42] in *E. coli*, and Rex in *Thermotoga maritima*[43]. In this work we predicted some new cases of regulation of CRISPR-Cas genes in the *Lactobacillales* genomes. The predicted operon *cas9-cas1-cas2-csn2* is preceded by a candidate CcpA-binding site in *S. agalactiae* and *S. mutans*, whereas in *S. agalactiae* and *S. equi* this operon has a candidate CodY-binding site in its promoter region. Another putative CRISPR-Cas operon, *cas5-cas8c-cas7-cas1-cas2*, is predicted to be regulated by CcpA in *S. equi* and *S. pyogenes.* These predicted regulatory interactions for CRISPR-Cas genes extend our understanding of regulatory mechanisms for bacterial immune systems.

### Evolution of regulons in Lactobacillales

#### Evolution of orthologous TF regulons

Among 102 TF regulons reconstructed in this work, 46 regulons have orthologous TFs in both studied lineages of the *Lactobacillales* (Additional file 5). We compared the deduced TFBS motifs for the orthologous TF regulons in the *Streptococcaceae* and *Lactobacillaceae* lineages and classified them into three categories (Additional file 9). Category I includes 23 TF regulons with binding motifs that are well conserved or slightly variable in two lineages. Category II contains 17 TF regulons with moderately different motifs (2 to 4 mismatches in the conserved motif positions). Category III has 6 remaining TF regulons with binding motifs that are substantially different between the *Streptococcaceae* and *Lactobacillaceae*. Remarkably, the category I is enriched by universal and highly conserved regulators. Thus, it includes all 5 regulons that are universal in both studied lineages and 10 regulons for TFs that are universal in the *Streptococcaceae* and have a mosaic distribution in the *Lactobacillaceae*. The category I contains the highest number of global regulons (CcpA, Rex) and mid-size regulons that control 3 or more target operons per genome (AdcR, ArgR, CtsR, GlnR, HrcA, MalR, MtaR, PerR and PurR). The remaining 13 regulons in this category are local, i.e. containing less than 3 target operons per genome. In contrast, the categories II and III contain respectively 94% and 100% of local regulons. These observations suggest that the conservation of TFBS motifs have a positive correlation with the regulon size. Similar correlation was previously reported for the *Staphylococcaceae* spp. [12].

Analysis of conservation of gene contents for orthologous TF regulons between the two studied lineages classified all regulons into three different categories. The first group contains 27 strictly conserved regulons that do not demonstrate any difference in their gene content or have only slight changes between the *Streptococcaceae* and *Lactobacillaceae* (*e.g*. an insertion of additional genes into target operons). The second group includes 16 regulons that have a common core of conserved genes supplemented by unique sets of peripheral genes that are substantially different between the *Streptococcaceae* and *Lactobacillaceae* . This group of partially conserved regulons includes the global regulons CcpA and Rex and several mid-size regulons (see below). The third group contains 3 orthologous regulons, MdxR, NiaR and NrtR, having completely different sets of target genes between the *Streptococcaceae* and *Lactobacillaceae* groups, although their corresponding biological roles are conserved between the studied groups. High diversity of the latter three regulons can be explained by redundancy of the respective metabolic systems and regulatory mechanisms. Thus, MdxR controls the maltose and maltodextrin utilization and the similar biological role was assigned to several other TF regulons in the Lactobacillales (see above). The phylogenetic distributions of two NAD metabolism regulons NiaR and NrtR have an overlapping pattern in many studied genomes (Additional file 5). In conclusion, we did not observe any correlation between the conservation of orthologous TF regulon structure and their cognate binding motifs in the analyzed taxonomic groups.

The 16 TF regulons with a common core and flexible set of peripheral genes between the *Streptococcaceae* and *Lactobacillaceae* groups can be classified into three subgroups: (i) regulons expanded in the *Streptococcaceae* (AdcR, BirA, CopR, GlnR, LacR and MntR); (ii) regulons expanded in the *Lactobacillaceae* (HrcA and ScrR), and (iii) regulons with different peripheral sets of genes in the *Streptococcaceae* and *Lactobacillaceae* (ArgR, CcpA, CtsR, MalR, NrdR, PerR, PurR and Rex). We prepose that different sets of peripheral genes in the above 16 regulons have appeared due to independent expansion of regulons in each lineage. A common core of the ArgR regulon is formed by genes for the arginine transport and biosynthesis. The periphery of this regulon in the *Streptococcaceae* consists of the *argR* gene and *arcABC* operon for arginine catabolism, whereas the extended part of the ArgR regulon in the *Lactobacillaceae* includes only carbamoyl-phosphate synthase (*carAB*). The PurR regulon in the *Streptococcaceae* is expanded by genes for metabolism of folate-associated one-carbon compounds, whereas in the *Lactobacillaceae* this regulon has additional genes for the adenine and guanine metabolism (*purB* and *guaB*) and ribose-phosphate pyrophosphokinase (*prsA*). Expansion of the PerR regulon in the *Streptococcaceae* affects genes for the iron and manganese homeostasis, whereas in the *Lactobacillaceae* PerR regulates several additional genes such as a NADH peroxidase. The CcpA regulon demonstrated the largest peripheral sets of genes in the studied lineages, 117 operons in the *Streptococcaceae* and 42 operons in the *Lactobacillaceae*. Noteworthy, the CcpA regulon in the *Streptococcaceae* is expanded by the CRISPR-Cas cassette genes (see above) and some virulence genes (the exfoliative toxin A gene *shetA* and the streptolysin S biosynthesis operon *sagABCEFGHI*), suggesting that this global regulon supplies the link between the carbohydrate utilization, virulence and anti-phage immunity.

#### Non-orthologous displacements of TF regulons

Functional analysis of reconstructed TF regulons in the *Lactobacillales* revealed that many biological processes are regulated by two or more non-orthologous TFs. These metabolic subsystems with redundant TF regulation include 8 distinct sugar utilization pathways (see above) and the zinc homeostasis that are controlled by 20 non-orthologous TFs (Additional file 5). In most cases, patterns of phylogenetic distribution of non-orthologous TFs controlling the same biological subsystem complement each other in the analyzed 30 genomes. Interestingly, there are only four cases in which the non-orthologous TFs belong to different protein families (CelR/CelQ, GntR1/GntR2, LacR/LacR2 and AdcR/Zur), whereas in all remaining cases the identified pairs of non-orthologous TFs belong to the same TF family and thus can be classified as cases of xenologous gene replacement [44]. The largest number of xenologous replacements of TFs was identified within the LacI family (Additional file 8).

### Interconnectivity in reconstructed TRNs

The cross talk between TF regulons can be identified by prediction of TFBSs for two or more TFs within the regulatory region of the same operon. Numerous target operons in the reconstructed TRNs are subject to regulation by multiple TFs. For instance, *S. preumoniae* has 38 target operons that share TFBSs for two or more TFs. Regulation of several target operons by multiple TFs is evolutionary conserved across a number of related genomes (Table 3).

**Table 3:** Examples of target operons regulation by multiple TFs

**Combinations of TFs**	**Examples**	
	**Target operon**	**Genome(s)**
***Quadruple regulation***		
AdcR, CcpA, CodY, Rex	*adhB1*	*S. agalactiae*, *S. dysgalactiae*
***Triple regulation***		
AdcR, CodY, Rex	*adhB1*	*S. equi*, *S. mitis*, *S. pyogenes*
MtaR, CmbR, HomR	*metEF*	*S. gallolyticus, S. gordonii, S. mutans, S. pneumoniae*
CcpA, MalR, MdxR	*malXCDA*	*L. plantarum*
CcpA, MalR3, MdxR	*nplT*	*L. plantarum*
CcpA, MalR, MdxR	*malT-mapA-pgmB*	*L. mesenteroides*
***Double regulation***		
CcpA, Rex	*adhE*	*S. mitis, S. sanguinis, L. acidophilus, L. brevis,L. johnsonii*
	*forT*	*S. dysgalactiae, S. equi, S. pyogenes, S. sanguinis, S. uberis*
CcpA, MalR	*ptsG-rgfB*	*S. agalactiae, S. equi, S. gordonii, S. mutans, S. uberis*
	*pulA*	*S. agalactiae, S. equi, S. gordonii, S. mutans, S. pyogenes*
HrcA, CtsR	*groSL*	all *Streptococcaceae, L. fermentum, L. reuteri, P. pentosaceus*
	*hrcA-grpE-dnaKJ*	*L. fermentum, L. plantarum, L. reuteri, L. sakei, P. pentosaceus*
CcpA, ScrR	*scrBR*	*S. gallolyticus, S. gordonii, L. acidophilus, L. johnsonii,*
CcpA, TreR	*trePA*	*S. dysgalactiae, S. pneumoniae, L. mesenteroidesm, O. oeni*
CcpA, GalR	*galKETRM*	*L. casei, L. rhamnosus*
	*lacLM*	*L. fermentum, L. plantarum, L. salivarius, P. pentosaceus*
CcpA, CodY	*livKHMGF*	*S. gordonii, S. mitis, S. sanguinis, S. suis, S. thermophilus*
CodY, GlnR	*gluQHMP*	*S. agalactiae, S. gallolyticus, S. mutans*
CcpA, FruR	*fruRBA*	*L. lactis, S. equi, S. suis, L. helveticus, L. sakei, P. pentosaceus*
PerR, MntR	*mntABC*	*S. equi, S. pyogenes, S. mutans, S. sanguinis, S. uberis*
CcpA, RegR	*hylD-ugl-hylEFG-ohl-regR*	*S. agalactiae, S. pyogenes*

Three global regulons, CcpA, CodY and Rex, often interconnect with each other and also with multiple local TF regulons. For example, the CcpA regulon overlaps with 31 TF regulons that control the carbohydrate utilization such as MalR, ScrR, TreR, GalR, FruR, and RegR. The CodY regulon overlaps with the GlnR and CmbR regulons that control the nitrogen metabolism and sulfur amino acid biosynthesis, respectively. A similar situation was previously observed in *S. aureus*, where the CcpA regulon overlaps with numerous local regulons for sugar utilization and the CodY regulon overlaps with amino acid metabolism regulons [12,45,46]. Similarly, co-regulation of two heat shock response regulons, HrcA and CtsR, was found both in the *Streptococcaceae* and *Lactobacillaceae* groups, and was also previously observed in *S. aureus*[12,47].

Autoregulation of TFs is a regular feature of the reconstructed regulons. An average portion of the *Lactobacillales* TFs with predicted control of their own expression is 72%. This index slightly varies between the analyzed genomes and is very close to the percentage of autoregulated TFs that was previously reported for *S. aureus*[12].

Multiple regulatory cascades between various TFs were detected in the studied genomes. For instance, CcpA regulates the *codY* gene and multiple genes encoding sugar utilization regulators, such as FruR, ScrR, TreR, and CelR. Among other identified cascades, CodY controls *glnR*, CmbR controls *homR* and PerR controls *mntR* in the *Streptococcaceae*, whereas CtsR controls *hrcA* in the *Lactobacillaceae*. Some cascades, such as the regulation of *fruR*, *galR*, *gutR*, *mtlR*, *scrR* and *treR* genes by CcpA, are conserved between the *Streptococcaceae* and *Lactobacillaceae* lineages, whereas the remaining cases of regulatory TF cascades are lineage specific. Comparison with the previously reconstructed regulatory network of *S. aureus*[12] showed that some of the identified *Lactobacillales* regulatory cascades are also conserved in the *Staphylococcaceae*. For instance, the CcpA-dependent regulation of *fruR*, *rbsR2*, *scrR* and *treR*, as well as the regulation of *glnR* by CodY and *hrcA* by CtsR, are conserved between the two groups. The conservation of cascades between distantly related genomes points to the importance of these regulatory interactions in the *Lactobacillales* regulatory networks.

## Conclusions

The knowledge-based bottom-up approach and comparative genomics techniques have been previously successively applied for reconstruction of bacterial TRNs in different groups of genomes [11,12]. Here we tentatively defined the reference collection of TF regulons in 30 *Lactobacillales* genomes comprised of 102 orthologous groups of TFs and ~4100 regulatory interactions (~140 per genome). The resulting regulatory network contains ~7500 regulated genes (~250 per genome) that are involved in sugar utilization, stress response, metal homeostasis and metabolisms of amino acids, fatty acids, nucleotides, and cofactors. We used a modified workflow for TRN reconstruction that is characterized by three main innovations: (1) analysis of two taxonomically related groups of genomes (the *Streptococcaceae* and *Lactobacillaceae*), (2) involvement of numerous experimental data from the literature about TF regulation in lactic acid bacteria, and (3) coordinated reconstruction of multiple TF regulons by a community of annotators using multi-user web interface of the RegPredict tool for regulon analysis [18]. By utilizing the semi-automatic workflow for regulon inference combined with manual curation and regulon annotation we described the largest reference collection of TF regulons in lactic acid bacteria do date. The collection also includes the previously uncharacterized regulons for 47 TFs that comprise ~1000 target genes (12 – 63 genes per genome). Thus, a significant number of predicted regulatory interactions and novel TF regulons await future experimental validation. During preparation of this manuscript, Bitoun *et al.* experimentally analyzed Rex regulon in the *S. mutans* UA159 [48] and confirmed Rex dependent regulation of 4 targets predicted in this work (*adhE*, *rex-guaA*, *ldh*, and *frdCT*).

Comparison of the inferred TRNs in the *Lactobacillales* genomes reveals interesting trends in the evolution of TRNs and individual TF regulons. First, we found a positive correlation between the TFBS motif conservation between the two lactic acid bacteria lineages and the distribution and conservation of the respective TF regulons. Second, we report that in the analyzed collection of *Lactobacillales* regulons non-orthologous displacements of TFs occur more often between structurally related TFs in comparison with regulators that belong to different protein families.

## Methods

Thirty complete genomes of *Lactobacillales* (Additional file 1) were downloaded from MicrobesOnline database [49]. Primary TF sets for each studied genome were extracted from P2TF (http://www.p2tf.org) database. Sigma factors and RNA binding proteins were excluded from the collections. Groups of orthologs were pre-counted as following. Initially, groups of orthologous proteins were constructed for every pair of TFs sets. All pairwise comparisons were done using BLASTP, and bidirectional best hits (BBHs) were identified if the protein sequences identity was more 50% and the aligned region was longer than 2/3 of the length of the shorter protein. If two paralogous genes from one genome were more similar to each other than to a BBH partner from another genome, both paralogs were added to the same orthology cluster. Finally, all orthologous clusters containing common genes were joined together. The clusters were formed using ad-hoc software written using Oracle RDBMS Express Edition (PL/SQL codes are available by request). TF families were assigned by analysis of protein domain structure using the following databases: CDD [50], Pfam [51], SMART [52], and MicrobesOnline Domain and Families [49].

For regulon reconstruction we used the previously established comparative genomics approach (reviewed in [10]) implemented in the RegPredict Web server (http://regpredict.lbl.gov) [18]. The approach includes inference of TFBSs, construction of nucleotide positional weight matrices (PWMs) for TFBSs motifs, and reconstruction of regulons in complete genomes on the basis of prediction of putative TFBSs in promoter gene regions. To take into account possible lineage specific changes in TFBSs motifs, we constructed individual PWMs for the *Streptococcaceae* and *Lactobacillaceae* taxonomic groups.

The three major workflows used for TF regulon reconstruction are (1) projection and expansion of previously known regulons from model *Lactobacillales* organisms, (2) projection of known regulons from model organisms belonging to another taxa, and (3) *ab initio* prediction of novel regulons (Figure 2).

In workflow 1, the projection and expansion of previously know TF regulons includes two slightly different workflows. In workflow 1a, both a set of regulated genes and TFBSs motif are known, whereas in workflow 1b, only a set of co-regulated genes is known from the collected experimental data. For previously known TFBSs motifs, a PWM was built and used for identification of additional sites in the analyzed genomes using the Run Profile tool in the RegPredict Web server. All novel true positive TFBSs were added to the training set and the updated PWM was constructed and further used for final regulon reconstruction. For regulons with originally unknown TFBS motifs, we collected a set of upstream regions of known TF-regulated genes and their orthologs and used this set for TFBS identification by the Discover Profile tool in the RegPredict. The TFBS motif discovery tool uses the expectation-maximization algorithm for clustering of all potential motifs with a specified symmetry (palindrome, direct or inverted repeat) and finally optimizes the inferred PWM. In ambiguous cases, putative regulatory elements were validated by phylogenetic footprinting [53] using multiple alignments for upstream non-coding regions of orthologous genes.

In workflow 2, the previously experimentally studied regulons in other model organisms from the Firmicutes phylum (*B. subtilis* or *S. aureus*) were projected to the *Lactobacillaceae* genomes. For TFBS identification, we used training sets of upstream regions of genes that are considered as orthologs to the TF-regulated genes from other model species outside of the *Lactobacillales* lineage.

Workflow 3 was used for *ab initio* prediction of novel TF regulons. Initially, the presumably co-regulated genes were predicted by the analysis of conserved gene neighborhoods around a putative TF gene. Upstream regions of presumably co-regulated genes extracted from multiple *Lactobacillales* genomes were used for identification of TFBSs and PWM construction as described above.

The obtained PWMs for known or predicted TFBS motifs were used for comparative genomics reconstruction of regulons in two groups of genomes, the *Lactobacillaceae* and *Streptococcaceae*, using the RegPredict Web server [18]. Each studied genome was scanned with the constructed PWMs using Run Profile tool in RegPredict. The threshold for site search was defined as a lowest score observed in the training set. The consistency check approach [10,54] and/or functional relatedness of candidate target operons were used to eliminate false positive TFBS predictions.

A community of annotators consisting of 18 undergraduate students from Hope College (Holland, MI) performed initial reconstruction of multiple TF regulons in the analyzed groups of genomes. This represented a coordinated annotation effort with expert curators in the context of a microbiology course. The resulting draft regulons underwent strict quality control to ensure accuracy of the reconstructions.

Functional gene annotations were uploaded from SEED [25], UniProt [55] and MicrobesOnline [49]. Multiple alignments of protein and DNA sequences were built by MUSCLE [56]. Phylogenetic trees were constructed using maximum likelihood algorithm implemented in PHYLIP package (v 3.69) [57] and visualized via Dendroscope tool [58]. Complete description of the reconstructed regulons including TFs, their target genes and operons, and associated TFBS were uploaded to the RegPrecise database (http://regprecise.lbl.gov) [27].

## Abbreviations

TF: Transcription factor; TFBS: Transcription factor binding site; TRN: Transcriptional regulatory network.

## Competing interests

The authors declare that they have no competing interests.

## Authors’ contributions

DARo and PSN conceived and designed the research project. DARa and DARo wrote the manuscript. DARa, DARo, and NVS performed comparative genomic analysis for reconstruction of regulons. DARo also provided the quality control as a curator. MDK computed orthologous clusters for TFs. AAB contributed to the development of the project, writing of the manuscript, and implemented the community annotation effort at Hope College. All authors read and approved the final manuscript.

## Supplementary Material

Additional file 1 **Phylogenetic tree of 30 studied *Lactobacillaceae *genomes.**- Description of data: Economic impact for each studied genome is shown in square brackets. The tree is based on approximately 78 universal prokaryotic proteins in the MicrobesOnline database: http://www.microbesonline.org/cgi-bin/speciesTree.cgi. Click here for file

Additional file 2 **Repertoire of DNA-binding transcriptional factors identified in 15 Streptococcaceae genomes.** Description of data: Orthologous groups are sorted by TF family and then by conservancy of each TF group. TFs with regulons reconstructed in this work are highlighted by light blue. Click here for file

Additional file 3 **Repertoire of DNA-binding transcriptional factors identified in 15 *Lactobacillaceae *genomes.** Description of data: Orthologous groups are sorted by TF family and then by conservancy of each TF group. TFs with regulons reconstructed in this work are highlighted by light blue. Click here for file

Additional file 4 **Distribution of predicted DNA binding transcription factors in studied *Lactobacillales *genomes.** Click here for file

Additional file 5 **Collection of TF regulons reconstructed in *Lactobacillales. *** Description of data: ^*a*^ Novel TF names introduced in this work are marked by asterisks. ^*b*^ Presence (+) or absence (−) of TFs orthologs. ^*c*^ Workflow 1, expansion and projection of a regulon previously characterized in model Lactobacillales organisms: (1a) TFBSs motif was known, (1b) TFBSs motif was predicted in present work; Workflow 2, projection of an orthologous regulon from *B. subtilis* or *S. aureus*; Workflow 3, *ab initio* regulon inference. Regulons previously studied in model organisms highlighted by green. Names of functional groups are highlighted in blue. Click here for file

Additional file 6 **Functional content, experimental evidences and conservation for reconstructed regulons in *Streptococcaceae. *** Description of data: Regulons are sorted by regulator names. Novel TF names introduced in this work are marked by asterisks. ‘Conservation of regulatory interaction’ column shows number of genomes with regulated gene/ operon (number of genomes having orthologs of operon). Click here for file

Additional file 7 **Functional content, experimental evidences and conservation for reconstructed regulons in *Lactobacillaceae. *** Description of data: Regulons are sorted by regulator names. Novel TF names introduced in this work are marked by asterisks. ‘Conservation of regulatory interaction’ column shows number of genomes with regulated operon (number of genomes having orthologs of operon). Click here for file

Additional file 8 **Analysis of LacI family TFs in the studied *Lactobacillales *genomes.** Click here for file

Additional file 9 **Comparison of predicted binding site motifs in *Streptococcaceae *and *Lactobacillaceae *genomes.** Description of data: ^1^ Sequences Logos were constructed using WebLogo package (http://weblogo.berkeley.edu/logo.cgi). ^2^ NS, number of binding site sequences used to construct Logo. ^3^ Category reflects a conservancy between TFBS motifs in *Streptococcaceae* and *Lactobacillaceae*: I, highly conserved motifs; II, moderately different motifs; III, substantially different motifs. ^4^ Number of genomes that contain this regulon. ^5^ Average number of target operon in regulon per genome. Click here for file
