# Supplementary material for: Genomic reconstruction of transcriptional regulatory networks in lactic acid bacteria
Source: BMC Genomics. 2013 Feb 12;14:94. doi: 10.1186/1471-2164-14-94 (PMC3616900; doi:10.1186/1471-2164-14-94)

Additional file 1

Phylogenetic tree of 30 studied *Lactobacillaceae* genomes. Economic impact for each studied genome is shown in square brackets. The tree is based on approximately 78 universal prokaryotic proteins in the MicrobesOnline database: http://www.microbesonline.org/cgi-bin/speciesTree.cgi.

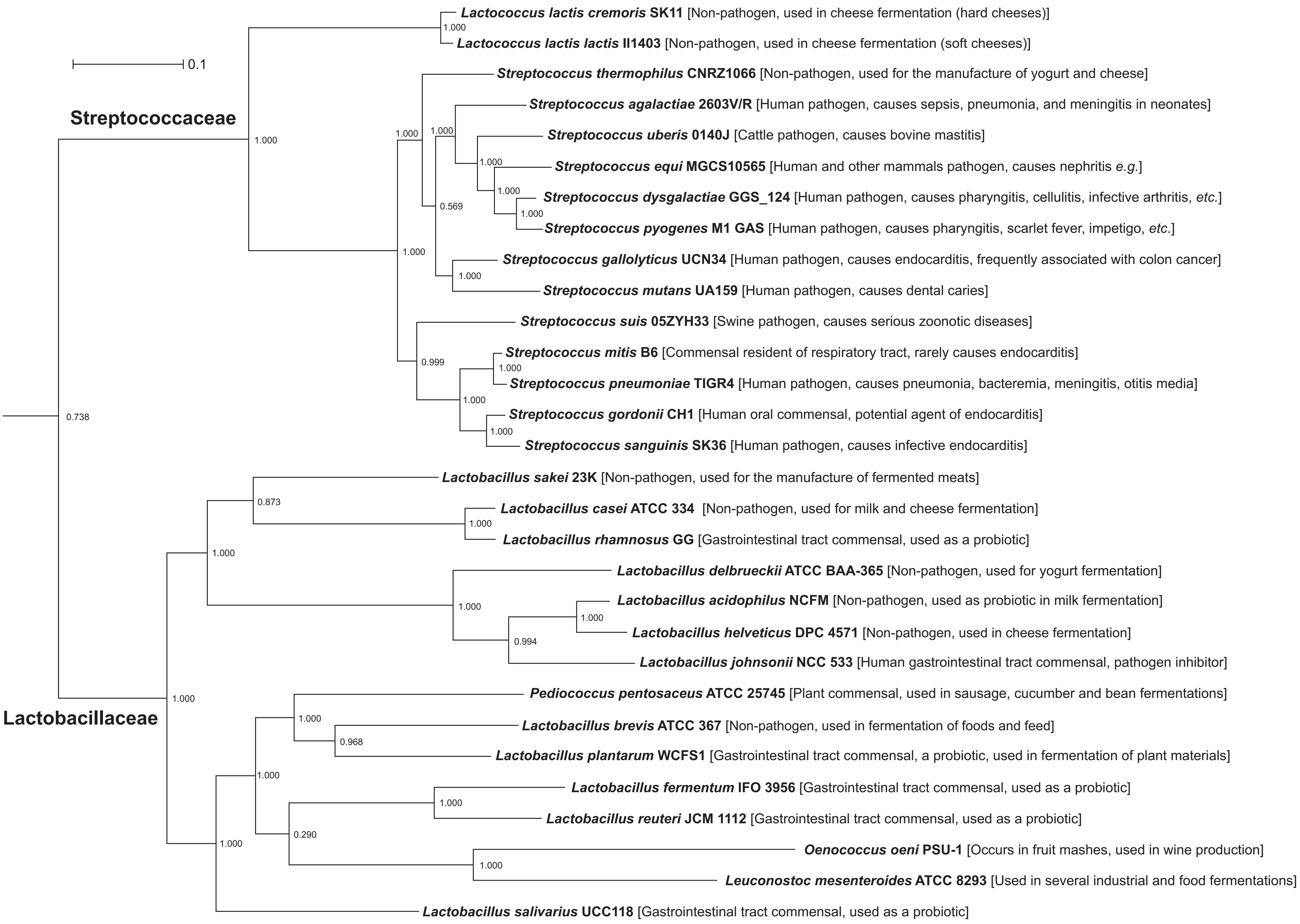

Supplement: Additional file 1 — Phylogenetic tree of 30 studied Lactobacillaceae genomes.- Description of data: Economic impact for each studied genome is shown in square brackets. The tree is based on approximately 78 universal prokaryotic proteins in the MicrobesOnline database: http://www.microbesonline.org/cgi-bin/speciesTree.cgi. [file 1471-2164-14-94-S1.pdf]
