# Supplementary material for: Genomic reconstruction of transcriptional regulatory networks in lactic acid bacteria
Source: BMC Genomics. 2013 Feb 12;14:94. doi: 10.1186/1471-2164-14-94 (PMC3616900; doi:10.1186/1471-2164-14-94)
Supplement: Additional file 8 — Analysis of LacI family TFs in the studied Lactobacillales genomes. [file 1471-2164-14-94-S8.pdf]

Additional file 8. **Analysis of LacI family TFs in the studied *Lactobacillales* genomes.**

The 26 of reconstructed carbohydrate metabolism regulons are controlled by TFs belonging to LacI protein family. Proteins of LacI family are well studied and broadly distributed in bacterial genomes [1-3]. In *Lactobacillales* species, 8 LacI regulators were previously analyzed experimentally [4-11]. Also, regulation by 12 LacI family proteins was previously studied in *L. plantarum* using comparative genomics approaches [12].

In this work we used known structural information about interactions between LacI family proteins for prediction of TFBS motifs for novel regulons. The new predicted TFBSs were compared with the known motif for related TFs and similarity between new and known TFBS motifs was used as an additional criterion for confirmation of the motif prediction accuracy.

Most of the reconstructed LacI family TF regulons control the peripheral carbon utilization pathways. For some pathway more than one LacI TF regulons were observed For example, two maltose/maltodextrin ABC transporters are controlled by two non-orthologous TFs from the LacI family in the *Streptococcaceae*. The *malEFG* operon is always regulated by MalR protein. The *malXCDA* operon is regulated by MalR in four *Streptococcus* spp. and under control of MalR2 in three other *Streptococcus* spp. Additionally, in majority of the studied genomes *malEFG* and/or *malXCDA* operons are also regulated by global catabolism regulator CcpA (Fig. S1).

TFBS motifs for 26 studied LacI family TFs are similar to each other and levels of similarity between different motifs is in a good agreement with the distances between cognate TFs (Fig. S2). Most of binding motifs for studied LacI family regulators are even palindromes of 16 to 20 b.p. Motifs for the GalR and LacR are exceptions and have structure of are odd palindromes. Such change of evenness for these motifs have been described previously [12], but still have not got appropriate explanation. The binding motif for CcpB orthologous group of TF conserved the structure of even palindrome but central CG nucleotides, typical for binding motifs of related TFs, was changed to GC. Previously the same features of binding motifs were observed for CcpB regulator from *B. subtilis* [13] which is orthologous to the *Lactobacillales* CcpB proteins. Functional distribution of LacI family TF regulons reflects the picture of the whole regulation of carbohydrate metabolism: the regulation of one metabolic pathway can be realized by different non-orthologous regulators even within the single LacI family of regulators.

Figure S1. **Regulation of genes for maltose and maltodextrin transporters in *Streptococcaceae* genomes.**
Genes are shown by arrows, the orthologous genes are shown by the same color. TF binding sites are shown by circles (see color labels in the top right color of the figure).

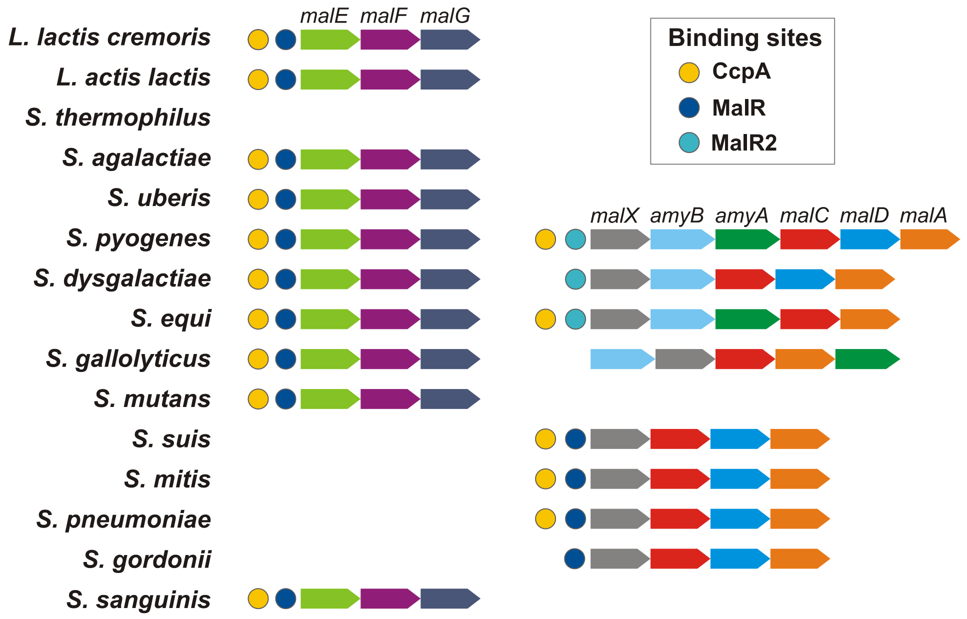

Figure S2. **Compressed phylogenetic tree for studied LacI family TF orthologous groups.**
Blue branches correspond to *Streptococcaceae* specific regulators; red branches correspond to *Lactobacillaceae* specific regulators; double blue and red branches are used for regulators present in both studied lineages. TFBS motifs logos for each TF orthologous row and biological roles for reconstructed regulons are shown on right. Different regulons with the same biological role are connected by angle lines.

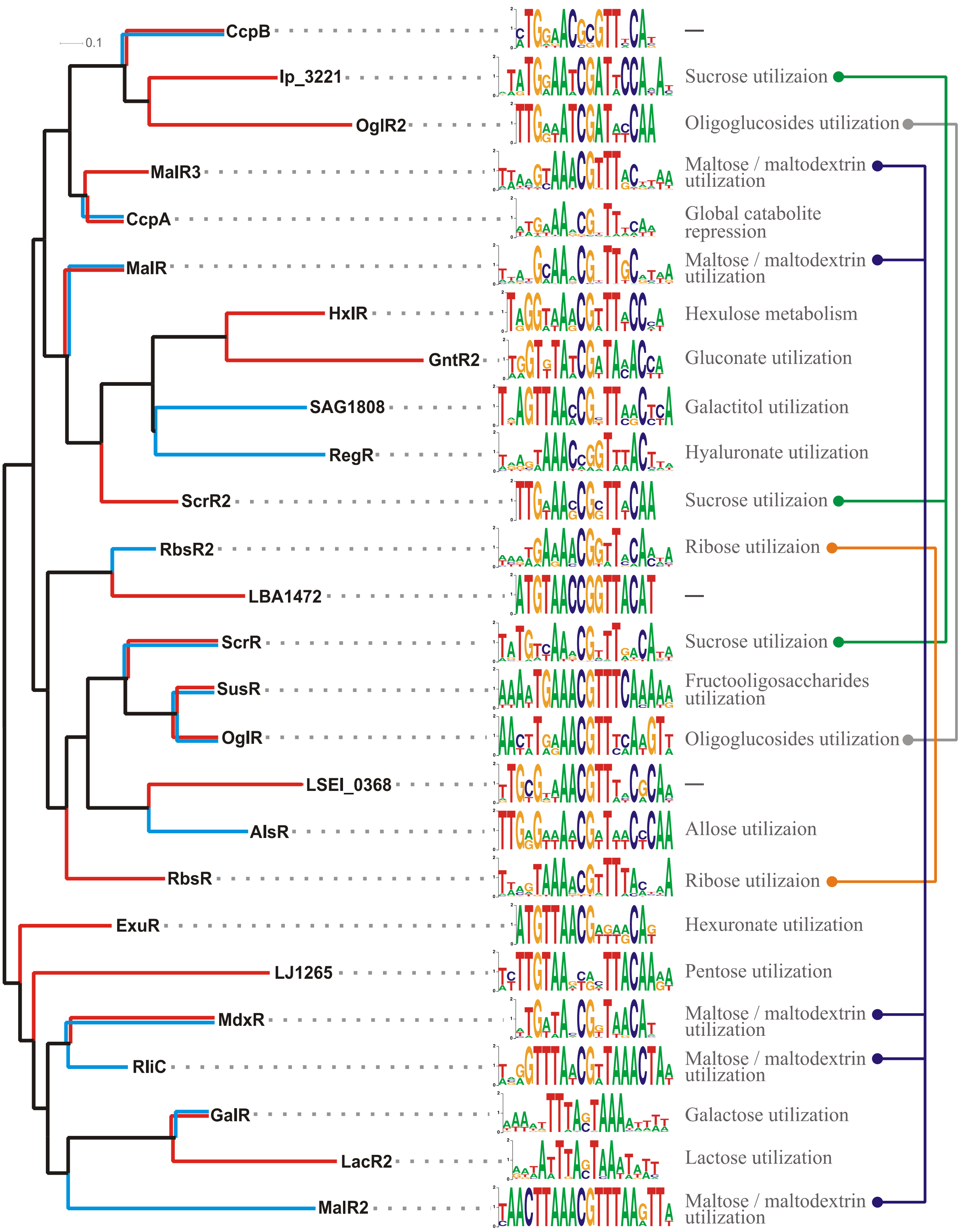

References

1. Milk L, Daber R, Lewis M: **Functional rules for lac repressor-operator associations and implications for protein-DNA interactions.** *Protein Sci* 2010, **19:**1162-1172.
2. Swint-Kruse L, Matthews KS: **Allostery in the LacI/GalR family: variations on a theme.** *Curr Opin Microbiol* 2009, **12:**129-137.
3. Tungtur S, Parente DJ, Swint-Kruse L: **Functionally important positions can comprise the majority of a protein's architecture.** *Proteins* 2011, **79:**1589-1608.
4. Zheng L, Chen Z, Itzek A, Herzberg MC, Kreth J: **CcpA regulates biofilm formation and competence in *Streptococcus gordonii*.** *Mol Oral Microbiol* 2012, **27:**83-94.
5. Zotta T, Ricciardi A, Guidone A, Sacco M, Muscariello L, Mazzeo MF, Cacace G, Parente E: **Inactivation of *ccpA* and aeration affect growth, metabolite production and stress tolerance in *Lactobacillus plantarum* WCFS1.** *Int J Food Microbiol* 2012.
6. Boucher I, Vadeboncoeur C, Moineau S: **Characterization of genes involved in the metabolism of alpha-galactosides by *Lactococcus raffinolactis*.** *Appl Environ Microbiol* 2003, **69:**4049-4056.
7. Chapuy-Regaud S, Ogunniyi AD, Diallo N, Huet Y, Desnottes JF, Paton JC, Escaich S, Trombe MC: **RegR, a global LacI/GalR family regulator, modulates virulence and competence in *Streptococcus pneumoniae*.** *Infect Immun* 2003, **71:**2615-2625.
8. Lapierre L, Mollet B, Germond JE: **Regulation and adaptive evolution of lactose operon expression in *Lactobacillus delbrueckii*.** *J Bacteriol* 2002, **184:**928-935.
9. Andersson U, Radstrom P: **Physiological function of the maltose operon regulator, MalR, in *Lactococcus lactis*.** *BMC Microbiol* 2002, **2:**28.
10. Stentz R, Zagorec M: **Ribose utilization in *Lactobacillus sakei*: analysis of the regulation of the *rbs* operon and putative involvement of a new transporter.** *J Mol Microbiol Biotechnol* 1999, **1:**165-173.
11. Iyer R, Camilli A: **Sucrose metabolism contributes to *in vivo* fitness of *Streptococcus pneumoniae*.** *Mol Microbiol* 2007, **66:**1-13.
12. Francke C, Kerkhoven R, Wels M, Siezen RJ: **A generic approach to identify Transcription Factor-specific operator motifs; Inferences for LacI-family mediated regulation in *Lactobacillus plantarum* WCFS1.** *BMC Genomics* 2008, **9:**145.
13. Chauvaux S, Paulsen IT, Saier MH, Jr.: **CcpB, a novel transcription factor implicated in catabolite repression in *Bacillus subtilis*.** *J Bacteriol* 1998, **180:**491-497.
