# Supplementary material for: Genomic reconstruction of transcriptional regulatory networks in lactic acid bacteria
Source: BMC Genomics. 2013 Feb 12;14:94. doi: 10.1186/1471-2164-14-94 (PMC3616900; doi:10.1186/1471-2164-14-94)
Supplement: Additional file 9 — Comparison of predicted binding site motifs in Streptococcaceae and Lactobacillaceae genomes. Description of data: 1 Sequences Logos were constructed using WebLogo package (http://weblogo.berkeley.edu/logo.cgi). 2 NS, number of binding site sequences used to construct Logo. 3 Category reflects a conservancy between TFBS motifs in Streptococcaceae and Lactobacillaceae: I, highly conserved motifs; II, moderately different motifs; III, substantially different motifs. 4 Number of genomes that contain this regulon. 5 Average number of target operon in regulon per genome. [file 1471-2164-14-94-S9.pdf]

Additional file 9. **Comparison of predicted binding site motifs in *Streptococcaceae* and *Lactobacillaceae*.**

TFs	Predicted site motif in *Streptococcaceae*		Predicted site motif in *Lactobacillaceae*		Category [3]	Genomes [4]	Target operons [5]
	Motif sequence Logo [1]	NS [2]	Motif sequence Logo [1]	NS [2]			
**AdcR**	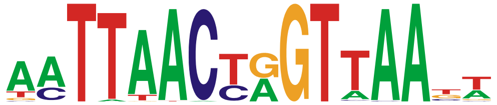	69	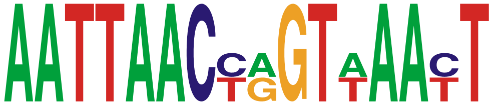	2	**I**	16	4.3
**AgaR2**	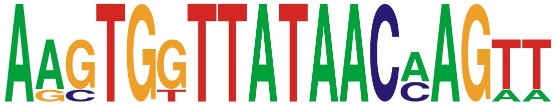	5	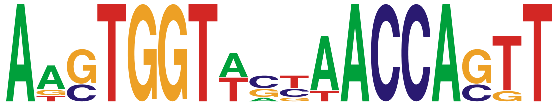	6	**I**	9	1.1
**ArgR**	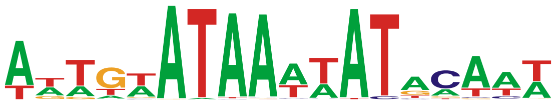	91	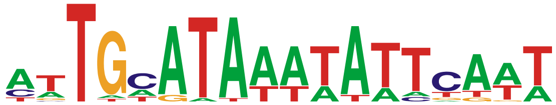	21	**I**	25	4.1
**BirA**	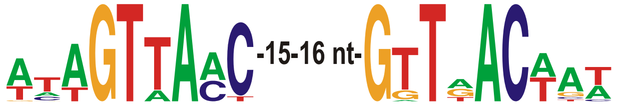	28	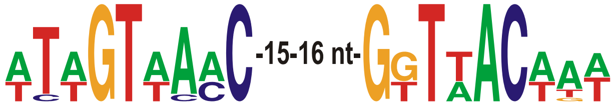	10	**I**	22	1.6
**BusR**	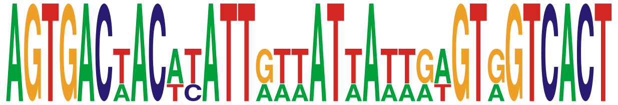	3	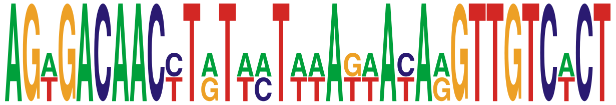	2	**I**	4	1.3
**CcpA**	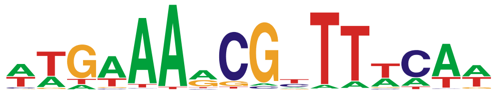	814	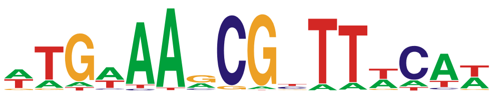	486	**I**	30	43.5
**CcpB**	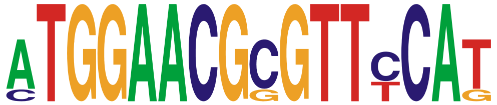	5	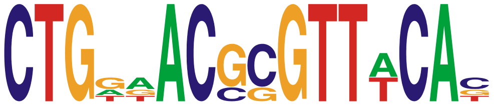	4	**II**	9	1.0
**CcpN**	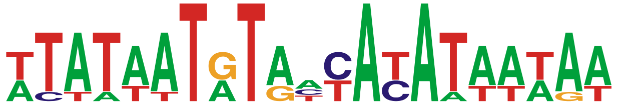	7	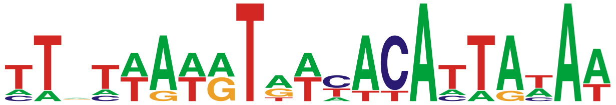	6	**II**	9	1.3
**CelR**	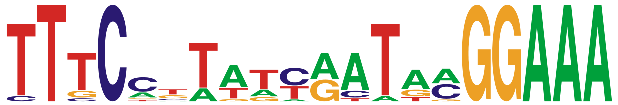	13	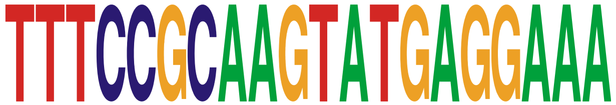	1	**II**	9	1.6

TFs	Predicted site motif in *Streptococcaceae*		Predicted site motif in *Lactobacillaceae*		Category [3]	Genomes [4]	Target operons [5]
	Motif sequence Logo [1]	NS [2]	Motif sequence Logo [1]	NS [2]			
CopR	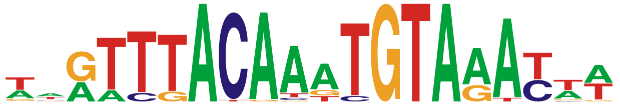	38	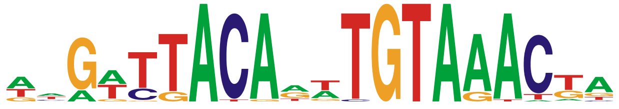	33	I	30	1.5
CtsR	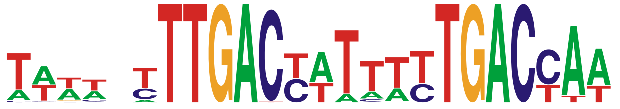	63	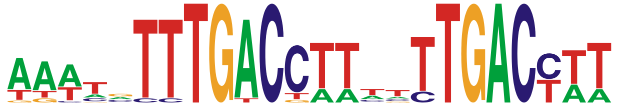	39	I	25	4.5
FabT	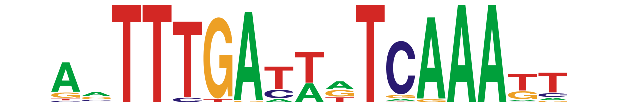	47	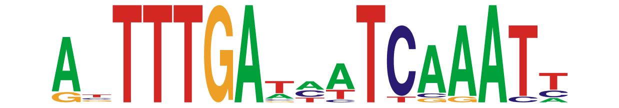	12	I	24	2.2
FlpA	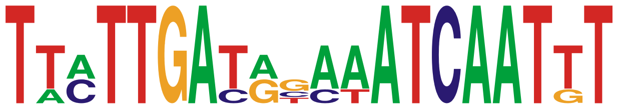	4	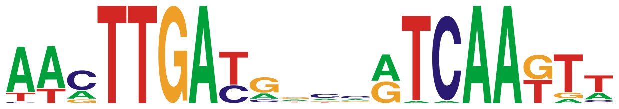	31	II	15	2.3
FlrR	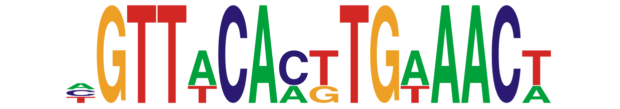	3	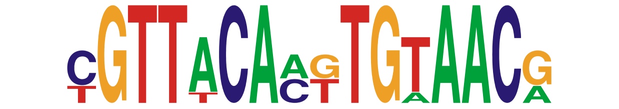	6	II	5	1.8
FruR	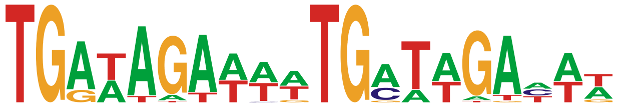	33	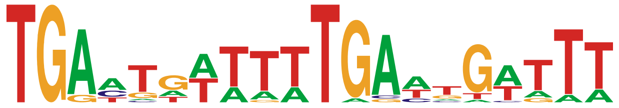	18	I	25	1.0
FucR	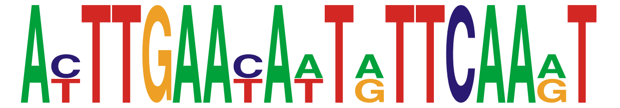	2	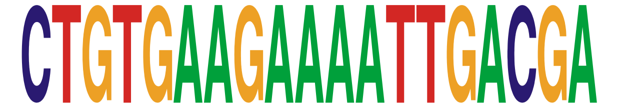	1	III	2	1.5
GalR	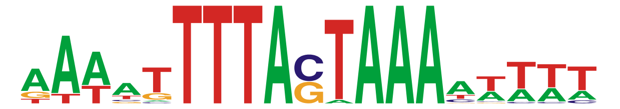	20	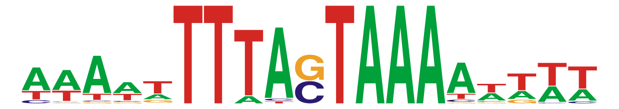	28	I	20	2.3
GlnR	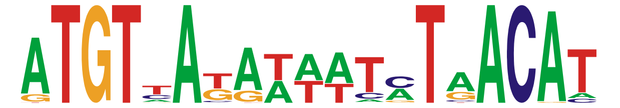	57	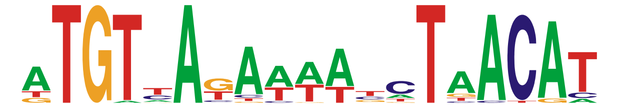	33	I	26	3.6
GntR1	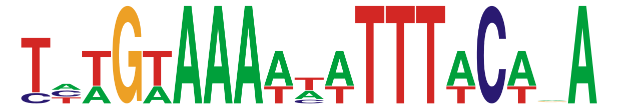	6	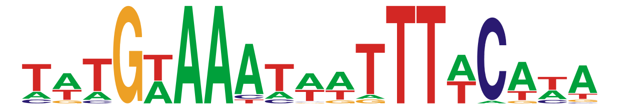	26	I	10	2.1

TFs	Predicted site motif in *Streptococcaceae*		Predicted site motif in *Lactobacillaceae*		Category [3]	Genomes [4]	Target operons [5]
	Motif sequence Logo [1]	NS [2]	Motif sequence Logo [1]	NS [2]			
**GutR**	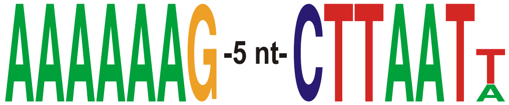	3	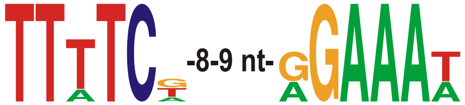	5	**III**	7	1.1
**HrcA**	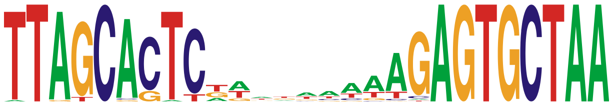	47	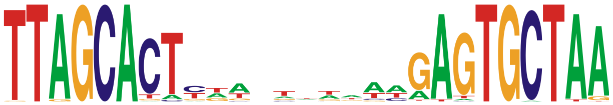	66	**I**	29	3.3
**LacR**	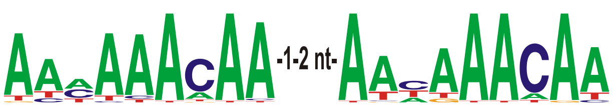	37	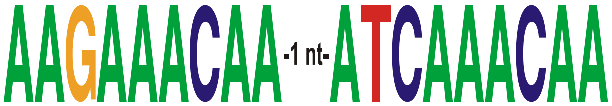	2	**II**	15	2.1
**MalR**	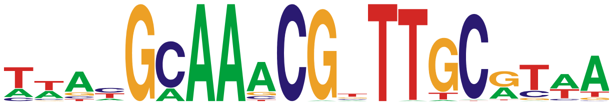	77	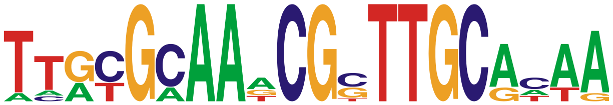	8	**I**	22	3.7
**MdxR**	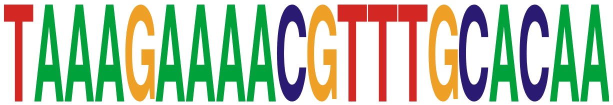	1	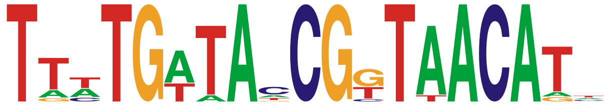	11	**II**	7	1.7
**MleR**	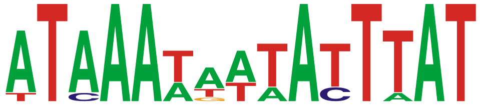	8	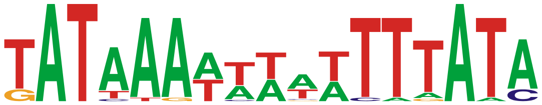	20	**I**	17	1.7
**MntR**	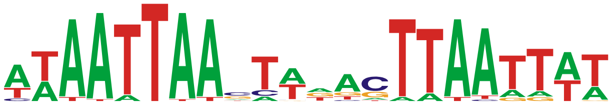	35	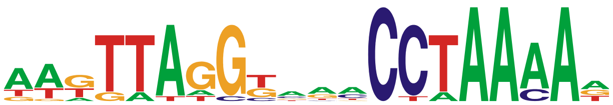	14	**III**	22	2.1
**MtaR**	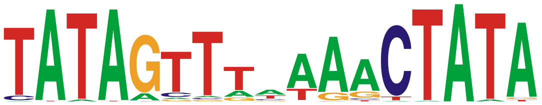	69	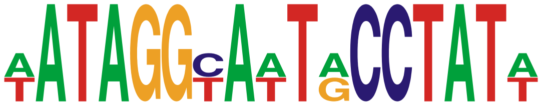	2	**II**	14	4.5
**MtlR**	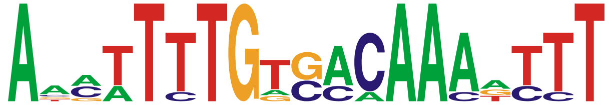	7	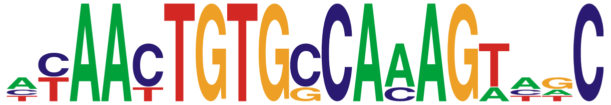	4	**III**	8	1.0
**MurR**	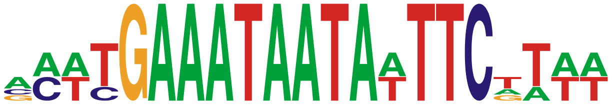	4	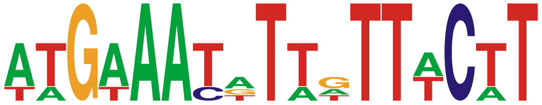	4	**II**	6	1.0

TFs	Predicted site motif in *Streptococcaceae*		Predicted site motif in *Lactobacillaceae*		Category [3]	Genomes [4]	Target operons [5]
	Motif sequence Logo [1]	NS [2]	Motif sequence Logo [1]	NS [2]			
**NagR**	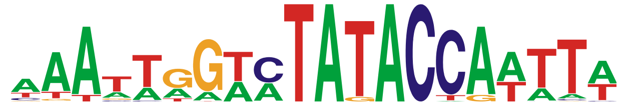	62	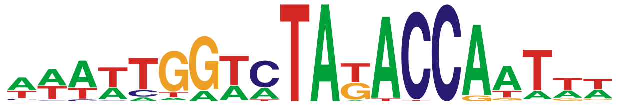	48	**I**	30	2.6
**NiaR**	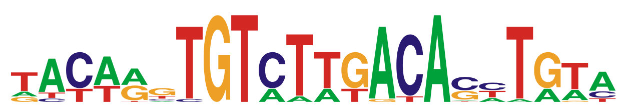	22	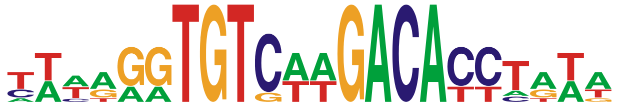	7	**I**	18	1.6
**NrdR**	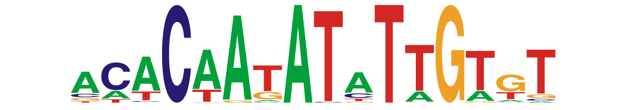	59	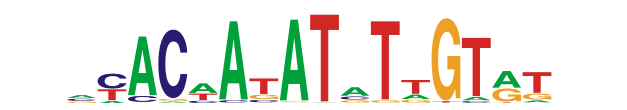	80	**I**	28	3.0
**NrtR**	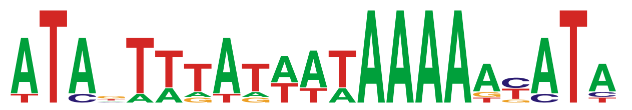	7	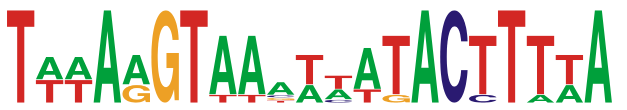	8	**III**	13	1.0
**OglR**	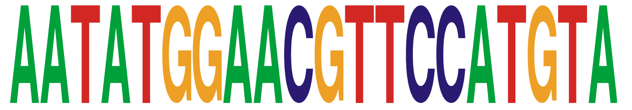	1	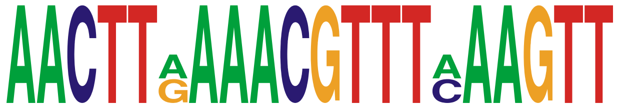	2	**II**	3	1.0
**PadR**	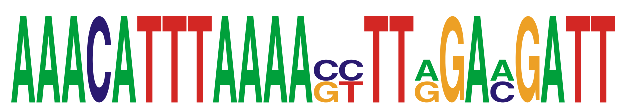	2	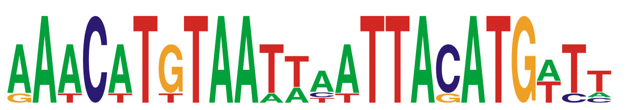	6	**II**	6	1.3
**PerR**	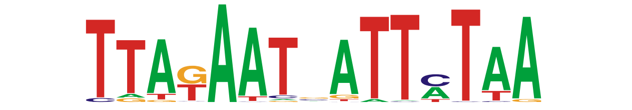	71	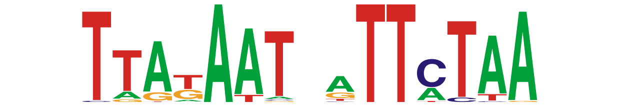	47	**I**	22	4.4
**PflR**	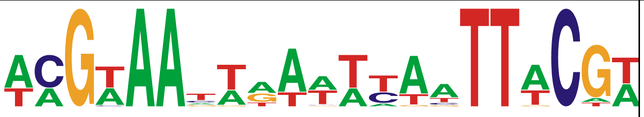	28	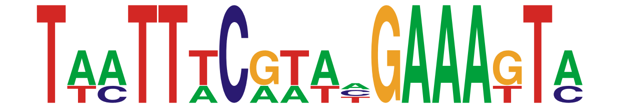	3	**III**	11	2.8
**PurR**	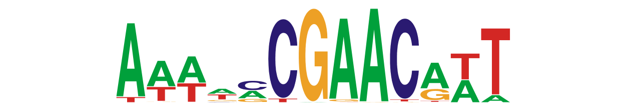	95	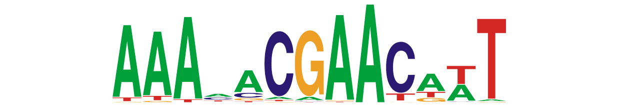	71	**I**	30	5.2
**Rex**	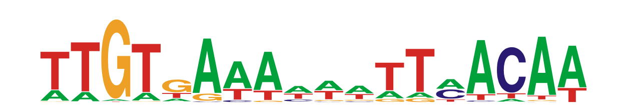	160	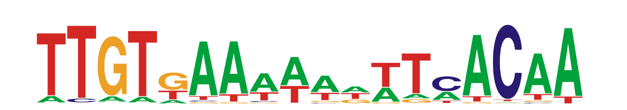	70	**I**	30	6.0

TFs	Predicted site motif in *Streptococcaceae*		Predicted site motif in *Lactobacillaceae*		Category [3]	Genomes [4]	Target operons [5]
	Motif sequence Logo [1]	NS [2]	Motif sequence Logo [1]	NS [2]			
ScrR	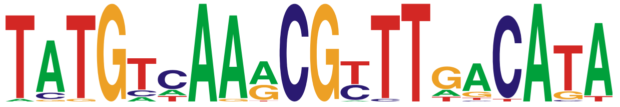	26	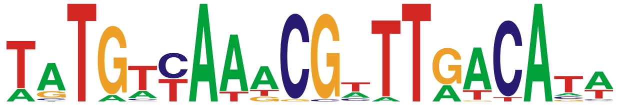	32	I	22	2.1
SusR	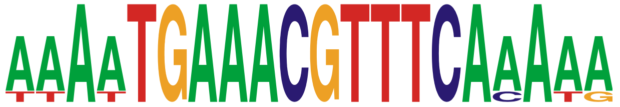	6	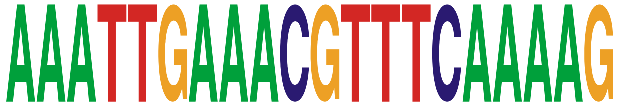	1	II	5	1.0
TagR	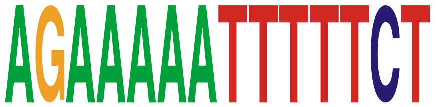	2	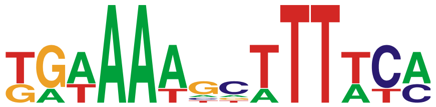	8	II	5	2.0
TreR	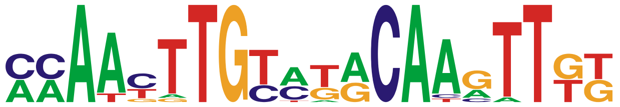	15	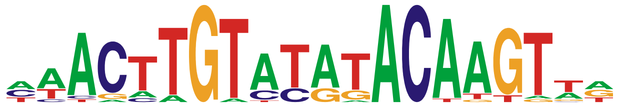	20	II	17	1.7
UxuR	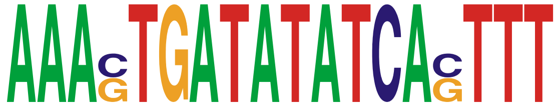	4	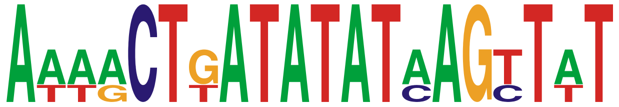	3	II	3	2.3
YwzG	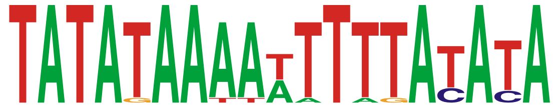	15	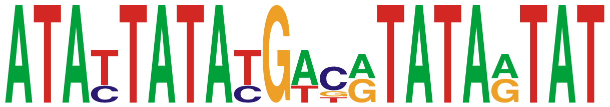	6	II	18	1.1
YxeR	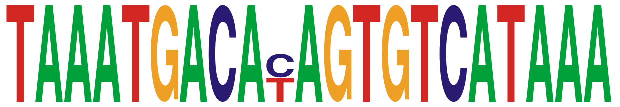	2	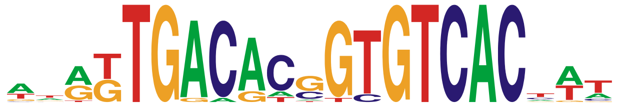	17	II	3	1.0

[1] Sequences Logos were constructed using WebLogo package (http://weblogo.berkeley.edu/logo.cgi)

[2] NS. number of binding site sequences used to construct Logo.

[3] Category reflects a conservancy between TFBS motifs in *Streptococcaceae* and *Lactobacillaceae*: I. highly conserved motifs; II. moderately different motifs; III. substantially different motifs.

[4] Number of genomes where current TF regulon was found.

[5] Average number of target operon in regulon per genome.
